# Transcriptomic Signatures of Neuronally Derived Extracellular Vesicles Reveal the Presence of Olfactory Receptors in Clinical Samples from Traumatic Brain Injury Patients

**DOI:** 10.3390/ijms25052777

**Published:** 2024-02-28

**Authors:** Manish Bhomia, Yanru Feng, Piper Deleon, Claudia S. Robertson, Firas Kobeissy, Kevin K. Wang, Barbara Knollmann-Ritschel

**Affiliations:** 1 Department of Pathology, Uniformed Services University of the Health Sciences, Bethesda, MD 20814, USA; yanru.feng.ctr@usuhs.edu (Y.F.); piper.deleon.ctr@usuhs.edu (P.D.); barbara.knollmann-ritschel@usuhs.edu (B.K.-R.); 2 Henry M. Jackson Foundation for the Advancement of Military Medicine, Bethesda, MD 20817, USA; 3 Department of Neurosurgery, Baylor College of Medicine, Houston, TX 77030, USA; claudiar@bcm.edu; 4 Department of Neurobiology, Morehouse School of Medicine, Atlanta, GA 30310, USA; fkobaissy@msm.edu (F.K.); kwang@msm.edu (K.K.W.)

**Keywords:** traumatic brain injury, exosomes, biomarkers

## Abstract

Traumatic brain injury (TBI) is defined as an injury to the brain by external forces which can lead to cellular damage and the disruption of normal central nervous system functions. The recently approved blood-based biomarkers GFAP and UCH-L1 can only detect injuries which are detectable on CT, and are not sensitive enough to diagnose milder injuries or concussion. Exosomes are small microvesicles which are released from the cell as a part of extracellular communication in normal as well as diseased states. The objective of this study was to identify the messenger RNA content of the exosomes released by injured neurons to identify new potential blood-based biomarkers for TBI. Human severe traumatic brain injury samples were used for this study. RNA was isolated from neuronal exosomes and total transcriptomic sequencing was performed. RNA sequencing data from neuronal exosomes isolated from serum showed mRNA transcripts of several neuronal genes. In particular, mRNAs of several olfactory receptor genes were present at elevated concentrations in the neuronal exosomes. Some of these genes were OR10A6, OR14A2, OR6F1, OR1B1, and OR1L1. RNA sequencing data from exosomes isolated from CSF showed a similar elevation of these olfactory receptors. We further validated the expression of these samples in serum samples of mild TBI patients, and a similar up-regulation of these olfactory receptors was observed. The data from these experiments suggest that damage to the neurons in the olfactory neuroepithelium as well as in the brain following a TBI may cause the release of mRNA from these receptors in the exosomes. Hence, olfactory receptors can be further explored as biomarkers for the diagnosis of TBI.

## 1. Introduction

Traumatic brain injury (TBI) is considered one of the major causes of disability and death worldwide, with approximately 1.7 million people affected just in the United States [1]. Over the past two decades, over 463,392 service members of the US military have been diagnosed with TBI, with the majority of brain injuries classified as mild TBI (mTBI) [2]. Among these injuries, cases caused by a blow to the head or from single or repetitive blast exposure are the most common causes of TBI. Clinically, TBI is diagnosed using the Glasgow Coma Scale (GCS) assessment tool followed by a computed tomography scan (CT). However, the GCS score can also reflect symptoms from non-TBIs or conditions, including intoxication from drugs or alcohol, sedative medications, or hypoxemia [3]. Additionally, the GCS score can underestimate mTBI cases [4]. CT scans also have several limitations, including poor visualization of the brain stem region, undetectable diffused axonal injury, need for a contrast agent to identify vascular injury, and exposure to radiation [5,6]. For these reasons, mTBI diagnosis presents a difficult clinical challenge, especially since many patients do not exhibit clinical symptoms of TBI.

Exosomes are small (40–100 nm) extracellular vesicles (EVs) that have been reported as key mediators of cell-to-cell signaling, diagnostic markers, and can be used as therapeutic interventions. Exosomes are derived from the invagination of late endosomes that result in the formation of intraluminal vesicles (ILVs), which then fuse with the plasma membrane, leading to the release of ILVs, now known as exosomes. Exosomes carry a variety of cargo, including DNA, RNA, proteins, microRNAs, and cell-free DNA [7]. Recent studies have suggested that the molecular signatures within exosomes potentially reflect pathobiological processes specific to their cell of origin. Furthermore, due to their small size, exosomes have been found to pass through the blood–brain barrier (BBB) and enter peripheral circulation, which makes them an attractive candidate for diagnostic markers.

Exosomes have been shown to be involved in cell-to-cell communication. Exosomes were identified to play a critical role in communication between the neuronal cells and astroglia in the central nervous system (CNS) [8]. Secondary injury, which follows a primary insult, is often characterized by microglial activation, astrogliosis, oxidative stress, proapoptotic gene expression, and calcium-mediated cellular damage [9]. Exosomes have been shown to play a critical role in the development and maintenance of neuroinflammation. In Alzheimer’s disease (AD), exosomes are reported to carry and transmit pathogenic proteins that lead to progression of the disease [10]. In cell culture studies, activated primary astrocytes have been shown to release exosomes with differentially expressed miRNAs that can potentially mediate the inflammatory response in the CNS [11].

Exosomes derived from neuronal cells have been explored for their utility in the diagnosis of several neurodegenerative conditions, as well as to understand the pathological role that they play in these disorders. Several studies have highlighted the importance of the cargo of neuronal exosomes in the diagnosis of AD, Parkinson’s disease (PD), and stroke [12,13,14]. Exosomal proteins and miRNAs have been reported as biomarkers to detect a TBI; however, the role of neuronally derived exosomes has not been studied in detail [15]. Moreover, the role and content of the exosomal transcriptome has not yet been reported for TBIs. In this study, our goal was to isolate neuronal exosomes derived from the serum of patients with severe TBI and characterize the transcriptome of neuronally derived exosomes. The transcriptome of the neuronal exosome can help identify novel biomarkers for TBI diagnosis and prognosis, and can also be helpful in determining the underlying pathological state of the CNS after an injury.

## 2. Results

### 2.1. Enrichment of Neuronal Extracellular Vesicles from Serum Samples of Patients with Severe TBI

**Figure 1:**
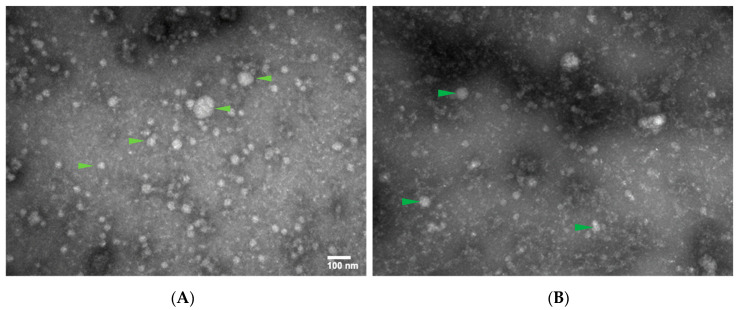
Detection of EVs from serum samples (scale bar = 100 nm). (**A**) Electron microscopy image of samples after total EV isolation (**B**) Electron microscopy image of samples after neuronal EV isolation.

Serum samples from patients with severe TBI (N = 24) were used for this study. The patient demographics are described in Table 1. Briefly, serum samples from three patients were pooled together to isolated total EVs in order to derive a better yield. EVs of neuronal origin were enriched using L1-CAM-based immunoprecipitation, as previously described. Electron microscopy (EM) analysis was performed to identify and validate the presence of EVs that were obtained using these methods. EM images showed the presence of EVs of varying size, 40–100 nm, highlighted with green arrows(Figure 1). EM images of L1-CAM enrichment showed a reduced concentration of EVs compared with total EVs (Figure 1A,B). The isolation of EV was performed using polymer-based precipitation; therefore, we had EVs of different sizes, as indicated Figure 1. Nevertheless, these results confirmed the presence of EVs after L1-CAM enrichment for further gene expression studies.

### 2.2. Sequencing of Neuronally Derived EV RNA

Total RNA isolated from the neuronally derived EVs was used for next-generation sequencing (NGS). We obtained a minimum of 100 million reads per sample, and more than 75% of the reads were mapped to the human genome (Table 1, Tables S1–S4 and Figure S1). Two-dimensional principal component analysis (PCA) scatter plots with convex hulls were drawn around two distinct groups: “STBI” and “control”. The percentages of variance captured by the PCA axes “PC1” and “PC2” were 15.06% and 11.94%, respectively (27% total) (Figure S2).

### 2.3. Differential Gene Expression

**Figure 2:**
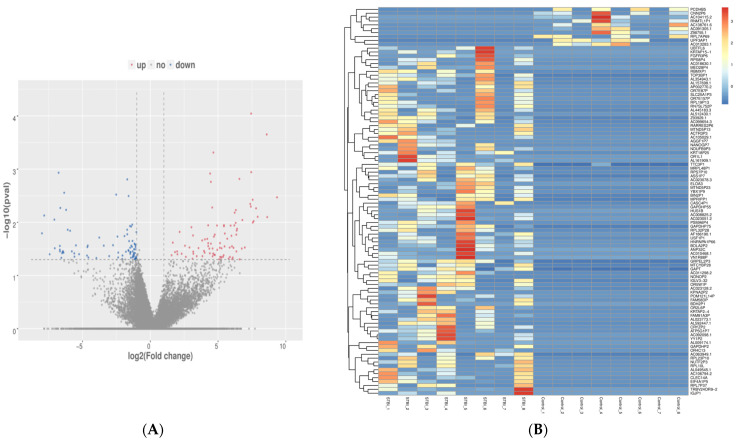
Differentially expressed mRNA. (**A**) Fold change against statistical significance. Red spots represent up-regulated genes with significant differential expression; blue spots are down-regulated genes; and gray spots are genes with non-differential expression. Dotted line represents the threshold for level of significance (*p* < 0.05) (**B**) Heat map of gene expression data from neuronally derived EVs. Heat plot depicts 99 differentially expressed mRNA genes. Each column represents one sample; each row represents one gene.

To identify the differentially expressed genes (DEGs) of mRNA, we created a volcano plot with statistical significance (Figure 2A). The plot was constructed using a p-value boundary of 0.050 and a fold-change of 2. In total, 99 mRNA DEGs with the highest expression were considered (10 down-regulated and 89 up-regulated) (Table 1). The heat map diagram showed the clustering of mRNA genes and samples, and revealed six olfactory receptor genes—OR4C13, OR7E87P, OR7E157P, OR5W1P, OR2L6P, and OR1L1—with up-regulated expression only in sTBI controls (Figure 2B).

### 2.4. GO and KEGG Pathway Analysis

**Figure 3:**
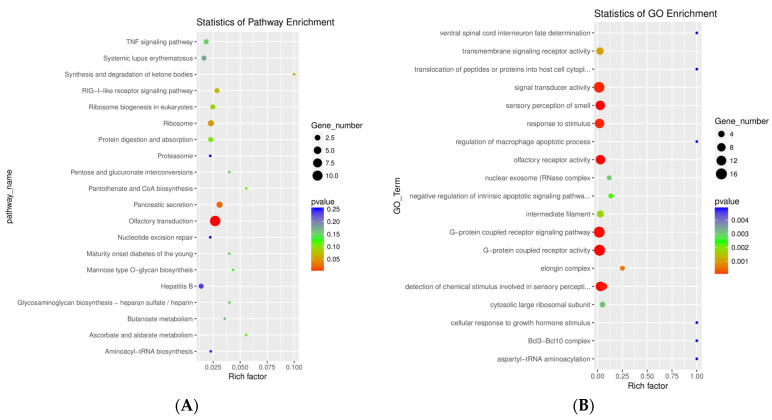
Bubble plots of KEGG and GO mRNA gene enrichment analysis. (**A**) KEGG analysis showing olfactory transduction pathway as highly enriched. (**B**) GO analysis showing olfactory receptor activity as one of the most significant.

Analysis of Kyoto Encyclopedia of Genes and Genomes (KEGG) and Gene Ontology (GO) revealed olfactory receptor activity as one of the most significant. KEGG pathway analysis revealed that the genes were highly enriched in olfactory transduction (Figure 3A). GO analysis showed that the mRNA genes took part in many biological processes, such as signal transduction activity, sensory perception of smell, response to stimulus, olfactory receptor activity, G-coupled protein signaling and receptor activity, and the detection of chemical stimulus involved in sensory perception (Figure 3B, Tables S5 and S6).

### 2.5. Validation of Olfactory Receptor Expression

**Figure 4:**
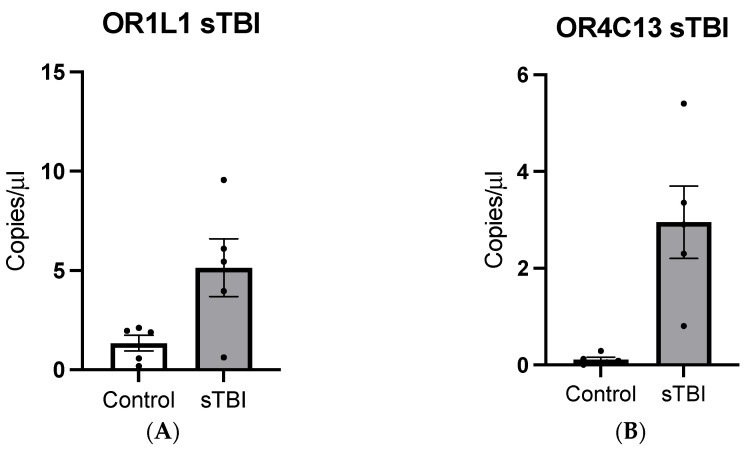
Expression of olfactory receptor genes in sTBI and non-TBI control samples. (**A**) OR1L1 expression in sTBI and non-TBI controls (*p* = 0.0359, N = 5). (**B**) OR4C13 expression in sTBI and non-TBI controls (*p* = 0.0053, N = 5).

**Figure 5:**
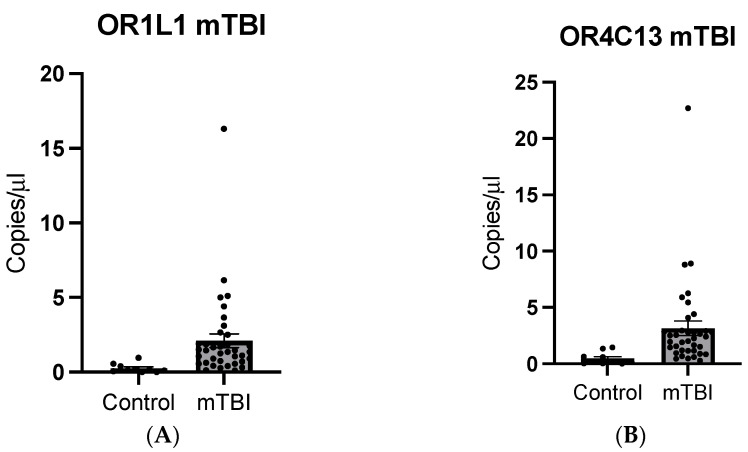
Expression of olfactory receptor genes OR1L1 and OR4C13. (**A**) OR1L1 expression in mTBI and non-TBI control samples (*p* < 0.001, N = 5). (**B**) OR4C13 expression in mTBI and non-TBI control samples (*p* < 0.001, N = 37 (mTBI), n = 10 (controls)).

The expressions of OR1L1 and OR4C13 were validated through droplet digital PCR (DDPCR) with sTBI samples (N = 5). Average expression of OR1L1 in sTBI samples revealed a greater concentration relative to the expression in non-TBI controls (Figure 4A; *p* = 0.0359, SEM = 1.508). DDPCR analysis also revealed a significantly higher expression of OR4C13 in sTBI samples compared with controls (Figure 4B; *p* = 0.0053, SEM = 0.6659). Expressions of the receptors were also examined in mTBI samples through DDPCR. The expression of OR1L1 in mTBI samples was greater than non-TBI controls (Figure 5A; *p* < 0.001, SEM = 0.4724). DDPCR analysis on the expression of OR4C13 in mTBI samples also revealed higher expression of the receptor in TBI samples when compared with controls (Figure 5B; *p* = 0.0003, SEM = 0.6659, N= 37 (mTBI) and N = 10 (controls)).

## 3. Discussion

TBI is a major public health problem worldwide. Globally, approximately 30–50 million people sustain a TBI every year, and these numbers are increasing every year [16]. The majority of TBI cases are categorized as mild, where most patients exhibit good recovery; however, a subset of these patients continue to have long-lasting neurological symptoms. Current clinical assessments of TBI involve the use of the GCS followed by a CT scan. The FDA has recently approved a blood-based biomarker to evaluate the need for a CT scan [17]; however, clinical translation has been limited to date. Many blood-based biomarkers have been explored for diagnosing a TBI, as well as understanding the resultant pathological state of the CNS. Recently, exosomes have been explored for biomarkers for several neurodegenerative disorders. Exosomal biomarkers have not been fully explored in cases of TBIs. In this study, our main objective was to evaluate the transcriptomic signature from neuronally derived EVs from a cohort of severe TBI patients.

Exosomes are synthesized by different cell types and are secreted into biofluids, such as blood, CSF, urine, and saliva. Additionally, exosomes are composed of various biomolecules that reflect the physiological state of the cell. This property of exosomes has made them attractive for the discovery of novel biomarkers for various neurodegenerative diseases including TBI [18]. Exosomes are known to carry cargoes including membrane proteins, cytoskeletal proteins, mRNA, and non-coding RNA such as lncRNA and miRNA. In this study, we focused on mRNA for the purpose of better understanding the transcriptome released from CNS after a TBI, specifically looking at the transcriptome of neuronally derived EVs.

Primary TBIs from original mechanical insults cause the deformation of tissue and degradation of cell membranes, while secondary injuries have biochemical and molecular cascades that further cell death, damage surrounding structures, and impair functionality. The onset of secondary injury after a TBI can occur around 48 h to days after the initial injury. Protein- and RNA-based biomarkers are well studied and reported in the literature, including from our group [11,19,20,21]. As our objective was to understand the transcriptomic signature of a sub-acute TBI, which could then be used as a sub-acute biomarker and help explain the pathological processes that are ongoing in the CNS, we used clinical specimens from patients with a severe TBI at 48 h post injury.

EVs have previously been studied for protein biomarkers, including GFAP, UCH-L1, NFL, and total tau, in a cohort of moderate to severe TBI patients, where they were shown to correlate with injury severity [15]. As described above, we isolated total EVs from pooled serum samples of severe TBI patients and then sub-fractionated neuronally derived EVs for our following studies. The electron microscopy results showed the presence of total EVs as well as neuronally derived EVs. Notably, the concentration of neuronally derived EVs was only a fraction of total EVs. The isolation of neuronally derived EVs was performed using the L1-CAM-based immunoprecipitation of total EVs, which has previously been shown to be a robust method for isolating neuronal EVs [22].

Transcriptomic profiling from neuronally derived EVs showed a significant modulation of 99 genes, among which 89 genes were found to be up-regulated and 10 genes were down-regulated in TBI samples when compared with control samples. Among these, several genes were found to be associated with cytokine signaling, apoptosis, and signal transduction. Interestingly, we found several full-length mRNAs for genes that were associated with olfactory signal transduction; these genes included OR4C13, OR7E87P, OR7E157P, OR5W1P, OR2L6P, and OR1L1. GO and KEGG analysis of the samples revealed that the olfactory transduction pathway and olfactory receptor activity were highly significant. Interestingly, the presence of olfactory receptor messenger RNA was only observed in neuronally derived EVs. Furthermore, many gene transcripts were also significantly up-regulated, demonstrating the presence of genes related to inflammation and olfaction. To the best of our knowledge, this is the first report to identify the presence of olfactory receptors in neuronal EVs in TBI patients. The presence of olfactory receptors in the sub-acute phase of injury may suggest an impaired sensory system after a TBI, and could be further explored as a potential biomarker for sub-acute TBI.

Additionally, we wanted to extrapolate our findings to mild TBI patients at an earlier timepoint after the injury to evaluate whether olfactory receptors can be detected in serum after a mild TBI. Therefore, we selected a patient population with mild TBIs and compared them with healthy controls using DDPCR. The samples from the mTBI group were collected between 4 and 24 h post injury. We evaluated the expression of OR1L1 and OR4C13, which were randomly selected from the group of olfactory receptors identified from previous experiments. Our results showed that OR1L1 and OR4C13 both were detected in mild TBI patients at a significantly elevated levels compared with healthy controls. Additionally, we validated the very low expression of these two genes in controls (Figure 5). Therefore, we conclude that olfactory receptors are present in serum after a mTBI, both at acute and sub-acute time points, and could therefore be further explored as a biomarker for concussive and sub-concussive injuries.

Olfactory impairment after TBI is relatively common and is dependent on the severity of the head trauma [23]. Studies researching olfactory dysfunction after head trauma have suggested that the site of injury may be a predictive factor [24]. Reports have shown significant correlations between olfactory dysfunction and skull base fractures, intracranial hemorrhage, and hematomas [25,26]. It has also been reported that a higher incidence of olfactory dysfunction has been noted in patients who suffer from injury to the occipital or frontal areas [24]. Additionally, there is a significantly stronger correlation between patients with post-traumatic amnesia of 5 min or more having olfactory dysfunction against those with post-traumatic amnesia of less than 5 min [27]. Olfactory receptors are predominantly found in the olfactory bulb; however, there is mounting evidence that shows the presence of these receptors in other regions of the mammalian brain, such as in the cerebral cortex, dopaminergic neurons of the substantia nigra, CA1 region of the hippocampus, and Purkinje cells [28]. Olfactory dysfunction can also occur due to injury to the central components of the olfaction system, such as the frontal and temporal lobes. Injury to the central component of the olfaction system usually does not lead to complete anosmia; rather, it can lead to impairments in olfaction recognition. This can be a direct result of the contusion, or may occur due to the inflammation that follows [29]. Therefore, it is possible that the NDEs secreted from the impacted cells in the region may contain olfactory signatures associated with them. Based on the information in the literature, it is possible that these olfactory receptors in NDE EVs may have originated from the olfactory epithelium as well as from other regions of the brain after a TBI. It has also been reported that these receptors on neurons, other than those present in the olfactory tract, are involved in more functions than just olfaction [28].

There are some limitations to this study, the first being the small sample size in the severe TBI validation cohort. The PCA plot was able to capture a 27% total variance from the first two principal components, which would improve with a larger sample size. Although the sample size was sufficient to determine statistical significance of the results, the experiments need to be replicated in a larger cohort of TBI patients to ensure their utility. Second, in this study, we randomly selected two olfactory receptors for validation. Further studies would require the validation of more receptors to identify their usefulness for TBI diagnosis. Finally, we have not studied the correlation of the presence of olfactory receptors with the severity of the injury or chronic outcomes due to the limited sample size. This could be studied in larger future studies.

## 4. Materials and Methods

### 4.1. Clinical Specimens

This study was reviewed and approved by the institutional review board of Uniformed Services University of the Health Sciences. The clinical serum samples for the severe TBI study were provided by Dr. Robertson and Dr. Wang. These samples were archived serum samples from a prior study conducted at Baylor College Medicine. Blood samples used in this study were originally collected as part of a previous clinical trial of erythropoietin (clinical trial.gov NCT00313716) [30]. The inclusion criteria for this trial included an age of at least 15 years, a Motor Glasgow Coma Scale score (mGCS) of 5 or less after resuscitation due to a closed head injury, and an availability to be enrolled within 6 h of injury. Exclusion criteria were a GCS of 3 with fixed, dilated pupils, penetrating trauma, pregnancy, life-threatening systemic injuries, and severe pre-existing disease. Blood samples for the trial were collected every 6 h for the first 24 h after injury, and then once daily until day 10 post-injury. The blood samples were centrifuged for 15 min at 1500× *g*, and the serum was removed and stored at −80 °C until analysis. Here, 24–48 h samples were used. For this study, the investigators received de-identified serum samples with no human identifiers. The demographic details of the severe TBI samples are listed in Table 2. The serum samples from mild TBI were acquired by the lead author from the biorepository of Center for Neuroscience and Regenerative Medicine at the Uniformed Services University of the Health Sciences. These samples were de-identified, and investigators only received the injury and demographic information (Table 3). Control sera used in this study were commercially acquired from bioIVT, with an equal representation of males and females.

### 4.2. Total EV Isolation

For total EV isolation, we used Exoquick reagent (System Biosciences Inc., Palo Alto, CA, USA), as recommended by the manufacturer. Briefly, we used 200 µL of pooled serum samples for EV isolation. In total, N = 24 sTBI samples were grouped into 8 groups of 3 samples in order to maximize the yield of EVs due to the limited availability of the serum samples. The samples were then centrifuged at 3000× *g* for 20 min, using an Eppendorf 5424 Microcentrifuge (Fisher Scientific, Waltham, MA, USA) to remove cellular debris. After Exoquick was added, the mixture was incubated for 30 min followed by centrifugation at 1500× *g* for 30 min at 4 °C. The EV pellet was then resuspended in 500 µL of nuclease-free water before neuronal EV enrichment.

### 4.3. L1-CAM Precipitation

To enrich neuronally derived EVs, the EV pellet was incubated with 4 µg of mouse anti-human biotin-conjugated CD-171 (L1-CAM) in 50 µL of 3% BSA made in PBS. Subsequently, 15 µL of streptavidin ultralink resin in a total volume of 40 µL of 3% BSA was added, then incubated before centrifugation and the addition of 0.1 M glycine-HCL. The supernatant was then transferred for use in RNA isolation.

### 4.4. RNA Isolation

RNA was isolated using the Sera IT kit (System Biosciences, CA, USA) following the manufacturer’s procedure: 350 µL of lysis buffer was added to the EVs before adding 200 µL of 100% ethanol, which was then transferred to a provided spin column and centrifuged at 13,000 rpm for 1 min. This was followed by repeated washes using 400 µL of wash buffer with columns centrifuged at 13,000 rpm for 1 min. RNA was then eluted with 25 µL of elution buffer. RNA quantities were measured using Nanodrop (Qiagen, Germantown, MD, USA).

### 4.5. cDNA Library Preparation and RNA Sequencing

Following RNA isolation, RNA fractions were fragmented using divalent cations under elevated temperatures. The cDNA libraries were prepared for whole-transcriptome sequencing using the TruSeq total RNA library preparation kit (Illumina Inc., San Diego, CA, USA). The average insert size for the pair-end library was 300 ± 50 bp; pair-end 2 × 150 bp sequencing was then performed on an Illumina Hiseq 4000 platform following the manufacturer’s protocol (Illumina). The RNA seq experiments were performed by a commercial vendor (LC Sciences Inc., Houston, TX, USA).

### 4.6. Bioinformatics Analysis

Program scripts Cutadapt and Perl were utilized in the removal of contaminated reads, low-quality bases, and undetermined bases. The quality of the sequences was verified using FastQC. Tophat and Bowtie were used to map reads to the human genome. Transcriptomes from samples were merged through Perl, StringTie and Ballgown were used to estimate the expression levels of all transcripts, and differential expression analyses of transcripts were performed using StringTie. The PCA was conducted in the R programming environment (R Core Team, 2023) using the G-median package and visualized with the ggplot2 package.

### 4.7. Olfactory Receptor Expression Validation Using Droplet Digital PCR

Mild TBI serum samples (N = 37) against non-TBI controls (N = 10), along with randomly selected sTBI serum samples against non-TBI controls (N = 5), were used for the validation of olfactory receptors OR4C13 and OR1L1 through droplet digital PCR (DDPCR). The RNA stock was first diluted at a concentration of 10 ng/µL and 4 µL of cellular RNA was used for reverse transcription reactions using Invitrogen SuperScript IV VILO Master Mix (Thermofisher Scientific, Waltham, MA, USA), as per the manufacturer’s protocol. DDPCR droplet generation was performed using the QX200 droplet generator (Bio-Rad, Hercules, CA, USA), as per the manufacturer’s protocol. Before droplet generation, a real-time PCR master mix was developed using DDPCR supermix for probes (Bio-Rad), nuclease-free water, mRNA specific 20× TaqMan primer (Thermofisher Scientific, Waltham, MA, USA), and droplet generation oil for probes (Bio-Rad, Hercules, CA, USA). Droplets were created using the droplet generator, and the plate was sealed after droplet formation using the PX1 PCR plate sealer (Bio-Rad, Hercules, CA, USA). PCRs were performed using the C1000 Touch thermal cycler (Bio-Rad), according to the manufacturer’s recommended thermal cycling conditions. The concentration of mRNA per reaction was analyzed using the QX200 droplet reader (Bio-Rad), and all reactions were duplicated. The collected DDPCR data were then analyzed using a parametric unpaired *t*-test with Welch’s correction in GraphPad Prism V9.

## 5. Conclusions

In conclusion, this is the first study to report the presence of olfactory receptors in neuronally derived EVs in mild to severe TBI patients at both acute and sub-acute phases of injury. Further studies should help determine whether these receptors can be used as biomarkers for concussive and sub-concussive injuries and other neurodegenerative diseases. Since these receptors are generally not detected in EVs, detection in blood samples could be useful in diagnosing TBIs.

## Figures and Tables

**Table 1:** Differential gene expression of neuronally derived EVs from serum samples of severe TBI patients compared with control.

Gene ID	Transcript ID	Gene Name	Regulation	Fold Change
MSTRG.1065088	ENST00000399586	*TTC3P1*	Up	21.32
MSTRG.772760	ENST00000318469	*GAPT*	Up	25.19
MSTRG.244357	ENST00000456608	*CASC4P1*	Up	173.81
MSTRG.148583	ENST00000527463	*AC023078.3*	Up	174.29
MSTRG.508652	ENST00000430812	*MTND5P23*	Up	386.52
MSTRG.593553	ENST00000420768	*TOP3BP1*	Up	Detected only in TBI
MSTRG.65467	ENST00000433910	*GAPDHP75*	Up	Detected only in TBI
MSTRG.920482	ENST00000448626	*GRPEL2P3*	Up	Detected only in TBI
MSTRG.1052920	ENST00000418200	*Z93929.1*	Up	Detected only in TBI
MSTRG.908115	ENST00000447087	*AC099654.3*	Up	Detected only in TBI
MSTRG.227545	ENST00000552900	*AC063949.1*	Up	Detected only in TBI
MSTRG.847293	ENST00000572103	*AL354943.1*	Up	Detected only in TBI
MSTRG.672063	ENST00000423174	*ASS1P7*	Up	Detected only in TBI
MSTRG.710875	ENST00000504996	*BIN2P1*	Up	Detected only in TBI
MSTRG.1062888	ENST00000415190	*AL157698.1*	Up	Detected only in TBI
MSTRG.762284	ENST00000603374	*AC008825.2*	Up	Detected only in TBI
MSTRG.513819	ENST00000420880	*AC023128.2*	Up	Detected only in TBI
MSTRG.288146	ENST00000342213	*CLEC14A*	Up	Detected only in TBI
MSTRG.622847	ENST00000452433	*MPRIPP1*	Up	Detected only in TBI
MSTRG.374937	ENST00000566728	*MTCYBP28*	Up	Detected only in TBI
MSTRG.585366	ENST00000451645	*USF1P1*	Up	Detected only in TBI
MSTRG.695441	ENST00000508931	*KRT18P25*	Up	Detected only in TBI
MSTRG.623962	ENST00000604904	*BOLA2P2*	Up	Detected only in TBI
MSTRG.324248	ENST00000423136	*GAPDHP55*	Up	Detected only in TBI
MSTRG.824871	ENST00000445390	*MRPL48P1*	Up	Detected only in TBI
MSTRG.21238	ENST00000414168	*AL445183.3*	Up	Detected only in TBI
MSTRG.716793	ENST00000504217	*CRYZP2*	Up	Detected only in TBI
MSTRG.593690	ENST00000390303	*IGLV3-32*	Up	Detected only in TBI
MSTRG.550449	ENST00000617989	*AC011298.2*	Up	Detected only in TBI
MSTRG.114674	ENST00000444398	*ATP5G1P7*	Up	Detected only in TBI
MSTRG.792237	ENST00000461022	*AC010468.1*	Up	Detected only in TBI
MSTRG.839571	ENST00000408004	*RBMXP1*	Up	Detected only in TBI
MSTRG.483037	ENST00000416539	*HNRNPA1P66*	Up	Detected only in TBI
MSTRG.929441	ENST00000429245	*AC092098.1*	Up	Detected only in TBI
MSTRG.853046	ENST00000401856	*AL049545.1*	Up	Detected only in TBI
MSTRG.949722	ENST00000483119	*RPL19P13*	Up	Detected only in TBI
MSTRG.1042496	ENST00000373686	*OR1L1*	Up	Detected only in TBI
MSTRG.88143	ENST00000416377	*OR2L6P*	Up	Detected only in TBI
MSTRG.959851	ENST00000468780	*RPL23P10*	Up	Detected only in TBI
MSTRG.48550	ENST00000456826	*FAM91A3P*	Up	Detected only in TBI
MSTRG.198076	ENST00000541376	*AC018630.1*	Up	Detected only in TBI
MSTRG.704071	ENST00000513046	*MTND5P13*	Up	Detected only in TBI
MSTRG.161814	ENST00000423705	*OR5W1P*	Up	Detected only in TBI
MSTRG.261252	ENST00000443577	*RPL32P28*	Up	Detected only in TBI
MSTRG.1009189	ENST00000397390	*AL161909.1*	Up	Detected only in TBI
ENSG00000229104	ENST00000438766	*YY1P2*	Up	Detected only in TBI
MSTRG.963864	ENST00000520250	*AC105029.1*	Up	Detected only in TBI
MSTRG.918147	ENST00000419546	*KPNA2P2*	Up	Detected only in TBI
MSTRG.843854	ENST00000406100	*POM121L14P*	Up	Detected only in TBI
MSTRG.1015387	ENST00000416632	*TRBV24OR9-2*	Up	Detected only in TBI
MSTRG.654115	ENST00000463779	*RN7SL752P*	Up	Detected only in TBI
MSTRG.203905	ENST00000541141	*AC023051.2*	Up	Detected only in TBI
MSTRG.169183	ENST00000543613	*AP002770.2*	Up	Detected only in TBI
MSTRG.1079250	ENST00000423985	*AL023773.1*	Up	Detected only in TBI
MSTRG.245206	ENST00000464254	*RPS7P10*	Up	Detected only in TBI
MSTRG.251588	ENST00000428062	*EIF4A1P5*	Up	Detected only in TBI
MSTRG.642943	ENST00000480448	*ACTR3P3*	Up	Detected only in TBI
MSTRG.482018	ENST00000414613	*NONOP2*	Up	Detected only in TBI
MSTRG.445754	ENST00000596787	*VN1R88P*	Up	Detected only in TBI
MSTRG.244415	ENST00000428076	*FAM58DP*	Up	Detected only in TBI
MSTRG.65174	ENST00000447592	*AL592447.1*	Up	Detected only in TBI
MSTRG.1077376	ENST00000426577	*AL009174.1*	Up	Detected only in TBI
MSTRG.1024304	ENST00000448540	*NUTF2P3*	Up	Detected only in TBI
MSTRG.556474	ENST00000432206	*GAPDHP2*	Up	Detected only in TBI
MSTRG.1058870	ENST00000433137	*MED28P4*	Up	Detected only in TBI
MSTRG.168438	ENST00000532397	*OR7E87P*	Up	Detected only in TBI
MSTRG.102959	ENST00000425095	*RPL7P37*	Up	Detected only in TBI
MSTRG.756566	ENST00000513713	*AC106794.2*	Up	Detected only in TBI
MSTRG.424620	ENST00000330682	*ELOA3*	Up	Detected only in TBI
MSTRG.391893	ENST00000394015	*KRTAP2-4*	Up	Detected only in TBI
MSTRG.997196	ENST00000517470	*AF186190.1*	Up	Detected only in TBI
MSTRG.741197	ENST00000512835	*ANP32C*	Up	Detected only in TBI
MSTRG.977904	ENST00000522682	*IGJP1*	Up	Detected only in TBI
MSTRG.584181	ENST00000334067	*KRTAP15-1*	Up	Detected only in TBI
MSTRG.79601	ENST00000441264	*YBX1P9*	Up	Detected only in TBI
MSTRG.858139	ENST00000602288	*BDH2P1*	Up	Detected only in TBI
MSTRG.134767	ENST00000454541	*RPS8P4*	Up	Detected only in TBI
MSTRG.494490	ENST00000477929	*UBTFL6*	Up	Detected only in TBI
MSTRG.308624	ENST00000554450	*NANOGP7*	Up	Detected only in TBI
MSTRG.291396	ENST00000298283	*RPL10L*	Up	Detected only in TBI
MSTRG.160760	ENST00000555099	*OR4C13*	Up	Detected only in TBI
MSTRG.273986	ENST00000603247	*PSMA6P4*	Up	Detected only in TBI
MSTRG.909669	ENST00000395415	*SLC25A1P3*	Up	Detected only in TBI
MSTRG.862400	ENST00000392575	*AL512430.1*	Up	Detected only in TBI
ENSG00000260994	ENST00000562369	*AGGF1P7*	Up	Detected only in TBI
MSTRG.948812	ENST00000494376	*OR7E157P*	Up	Detected only in TBI
MSTRG.820592	ENST00000380907	*HUS1B*	Up	Detected only in TBI
MSTRG.87795	ENST00000457012	*FGFR3P6*	Up	Detected only in TBI
MSTRG.360057	ENST00000567333	*RARRES2P6*	Up	Detected only in TBI
MSTRG.804116	ENST00000231134	*PCDHB5*	Down	0.01
MSTRG.617343	ENST00000439038	*CNN2P6*	Down	Detected only in control
MSTRG.434022	ENST00000479476	*AC091305.1*	Down	Detected only in control
MSTRG.386285	ENST00000583997	*AC138761.6*	Down	Detected only in control
MSTRG.819904	ENST00000523026	*AC104115.2*	Down	Detected only in control
MSTRG.859503	ENST00000407015	*Z98755.1*	Down	Detected only in control
MSTRG.384426	ENST00000413731	*UPF3AP1*	Down	Detected only in control
ENSG00000268483	ENST00000595306	*RPL7AP69*	Down	Detected only in control
MSTRG.97165	ENST00000454103	*RNMTL1P1*	Down	Detected only in control
ENSG00000225185	ENST00000557282	*AC013283.1*	Down	Detected only in control

**Table 2:** Demographic information of sTBI serum samples.

Demographic	sTBI (N = 24)
Sex	
Male	22
Female	2
Race	
White	3
Black	3
Hispanic	17
Asian	1
Other	0
Age at test, in years	
(Mean, SD, Range)	32.73 (12.28) 16–68
Mechanism	
Automobile accidents	19
Incidental Fall	4
Violence/Assault	1
EC Marshall CT Category	
M1	9
D2	10
D3	5
Month 6 GOS	
Severe Disability	13
Moderate Disability	3
Dead	4
Lost to follow-up	4
Vegetative State	0
ER GCS Score	
Severe	24

**Table 3:** Demographic information of mTBI serum samples.

Demographic	mTBI (N = 37)
Sex	
Male	26
Female	11
Age at test, in years	
(Mean, SD, Range)	44.11 (19.08) 19–87
Mechanism	
Automobile Accident	17
Incidental Fall	14
Other non-intentional injury	3
Violence/Assault	3
Post-Traumatic Amnesia	
Positive	33
Negative	4
CT Scan	
Positive	17
Negative	20

## Data Availability

All the data associated with this study are presented in the main manuscript and the Supplementary data section.

## Supplementary Materials

The supporting information can be downloaded at: https://www.mdpi.com/article/10.3390/ijms25052777/s1.
