# Supplementary material for: Transcriptomic Signatures of Neuronally Derived Extracellular Vesicles Reveal the Presence of Olfactory Receptors in Clinical Samples from Traumatic Brain Injury Patients
Source: Int J Mol Sci. 2024 Feb 28;25(5):2777. doi: 10.3390/ijms25052777 (PMC10931597; doi:10.3390/ijms25052777)
Supplement: Supplementary file 1 [file ijms-25-02777-s001.zip › ijms-2617078-supplementary.pdf]

Supplementary Figure and Tables.

Table S1. Quality control and statistics of reads

Sample	Raw Data		Valid Data		Valid Ratio(reads)	Q20%	Q30%	GC content%
	Read	Base	Read	Base				
Control_1	166205838	24.93G	161613252	24.24G	97.24	99.39	94.44	44
Control_2	160207896	24.03G	155849662	23.38G	97.28	99.44	95.11	44
Control_3	180642412	27.10G	176178340	26.43G	97.53	99.51	94.69	43
Control_4	139469322	20.92G	135755164	20.36G	97.34	99.48	94.73	48
Control_5	120342794	18.05G	117165130	17.57G	97.36	99.42	94.73	45
Control_6	180521446	27.08G	175614722	26.34G	97.28	99.23	94.19	43.5
Control_7	204566354	30.68G	199392580	29.91G	97.47	99.72	96.71	44
Control_8	139871038	20.98G	135636406	20.35G	96.97	99.29	95.73	44
STBI_1	129584956	19.44G	125393230	18.81G	96.77	98.64	93.22	46
STBI_2	162814002	24.42G	157451142	23.62G	96.71	98.71	94.12	44
STBI_3	140644900	21.10G	136306318	20.45G	96.92	98.91	94.5	45
STBI_4	133357446	20.00G	129248800	19.39G	96.92	98.68	92.97	46
STBI_5	143871490	21.58G	139460808	20.92G	96.93	99.29	92.88	46
STBI_6	158970820	23.85G	154087856	23.11G	96.93	99.58	95.6	45
STBI_7	171703474	25.76G	167155746	25.07G	97.35	99.69	96.19	44
STBI_8	165042378	24.76G	160028714	24.00G	96.96	99.41	95.42	45

Table S2. Mapping to genome

Sample	Valid reads	Mapped reads	Unique Mapped reads	Multi Mapped reads	PE Mapped reads	Reads map to sense strand	Reads map to antisense strand	Non-splice reads	Splice reads
Control_1	147847412	126472225 (85.54%)	106937837 (72.33%)	19534388 (13.21%)	89273488 (60.38%)	61200988 (41.39%)	61456112 (41.57%)	122187776 (82.64%)	469324 (0.32%)
Control_2	143211202	119068664 (83.14%)	104211954 (72.77%)	14856710 (10.37%)	69905494 (48.81%)	57539428 (40.18%)	57872570 (40.41%)	115048673 (80.33%)	363325 (0.25%)
Control_3	164639384	140481847 (85.33%)	121916225 (74.05%)	18565622 (11.28%)	86781274 (52.71%)	67910291 (41.25%)	68262013 (41.46%)	135683880 (82.41%)	488424 (0.30%)
Control_4	114714628	73430378 (64.01%)	64860458(56.54%)	8569920 (7.47%)	41732470 (36.38%)	35584360 (31.02%)	35837476 (31.24%)	71251354 (62.11%)	170482 (0.15%)
Control_5	105439778	79137827 (75.06%)	69302840 (65.73%)	9834987 (9.33%)	45171688 (42.84%)	38319315 (36.34%)	38530367 (36.54%)	76640565 (72.69%)	209117 (0.20%)
Control_6	162911330	140619712 (86.32%)	118568722 (72.78%)	22050990 (13.54%)	104009650 (63.84%)	68214639 (41.87%)	68456109 (42.02%)	136226184 (83.62%)	444564 (0.27%)
Control_7	186074406	149919288 (80.57%)	132705345 (71.32%)	17213943 (9.25%)	83815706 (45.04%)	72393810 (38.91%)	72793204 (39.12%)	144655518 (77.74%)	531496 (0.29%)
Control_8	124574732	97763810 (78.48%)	87089264 (69.91%)	10674546 (8.57%)	46550652 (37.37%)	47364384 (38.02%)	47584418 (38.20%)	94692359 (76.01%)	256443 (0.21%)
STBI_1	112572930	87622908 (77.84%)	76827515 (68.25%)	10795393 (9.59%)	50472938 (44.84%)	42517691 (37.77%)	42686769 (37.92%)	84957514 (75.47%)	246946 (0.22%)
STBI_2	143324992	118284440 (82.53%)	102781096 (71.71%)	15503344 (10.82%)	77109852 (53.80%)	57289100 (39.97%)	57488597 (40.11%)	114398105 (79.82%)	379592 (0.26%)
STBI_3	123551548	94661132 (76.62%)	82940161(67.13%)	11720971 (9.49%)	53992430 (43.70%)	45857134 (37.12%)	46050982 (37.27%)	91649081 (74.18%)	259035 (0.21%)
STBI_4	115936838	87067991 (75.10%)	76527126(66.01%)	10540865 (9.09%)	44089936 (38.03%)	42122735 (36.33%)	42335156 (36.52%)	84208703 (72.63%)	249188 (0.21%)
STBI_5	127168388	92212706 (72.51%)	78935164 (62.07%)	13277542 (10.44%)	62298968 (48.99%)	44715510 (35.16%)	44867075 (35.28%)	89309476 (70.23%)	273109 (0.21%)
STBI_6	139951168	107077413 (76.51%)	92033726 (65.76%)	15043687 (10.75%)	77320902 (55.25%)	51899977 (37.08%)	52070288 (37.21%)	103670611 (74.08%)	299654 (0.21%)
STBI_7	156213084	135642998 (86.83%)	115909870 (74.20%)	19733128 (12.63%)	95964978 (61.43%)	65499761 (41.93%)	65733128 (42.08%)	130682440 (83.66%)	550449 (0.35%)
STBI_8	145032926	121182442 (83.56%)	103755733 (71.54%)	17426709 (12.02%)	83483852 (57.56%)	58771381 (40.52%)	58962226 (40.65%)	117374117 (80.93%)	359490 (0.25%)

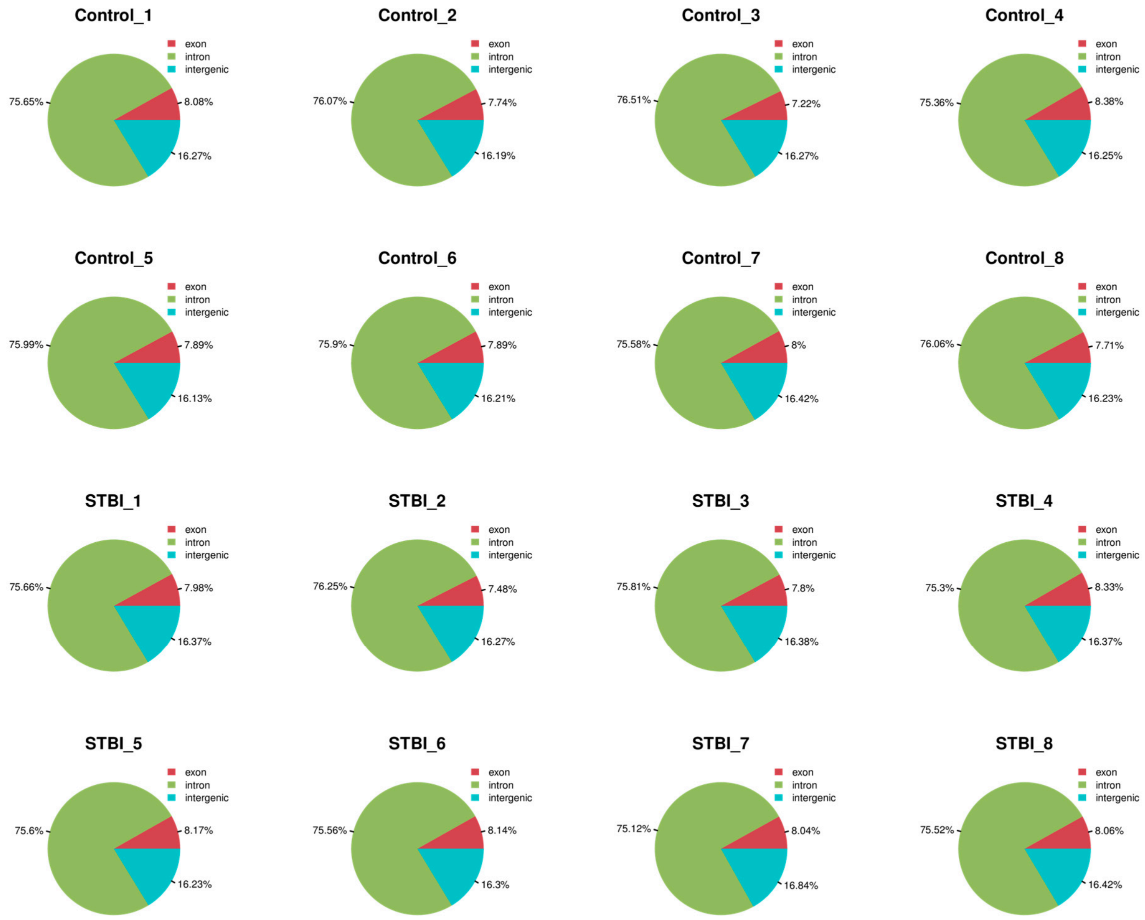

Figure S1. Statistics of mapped reads to genome

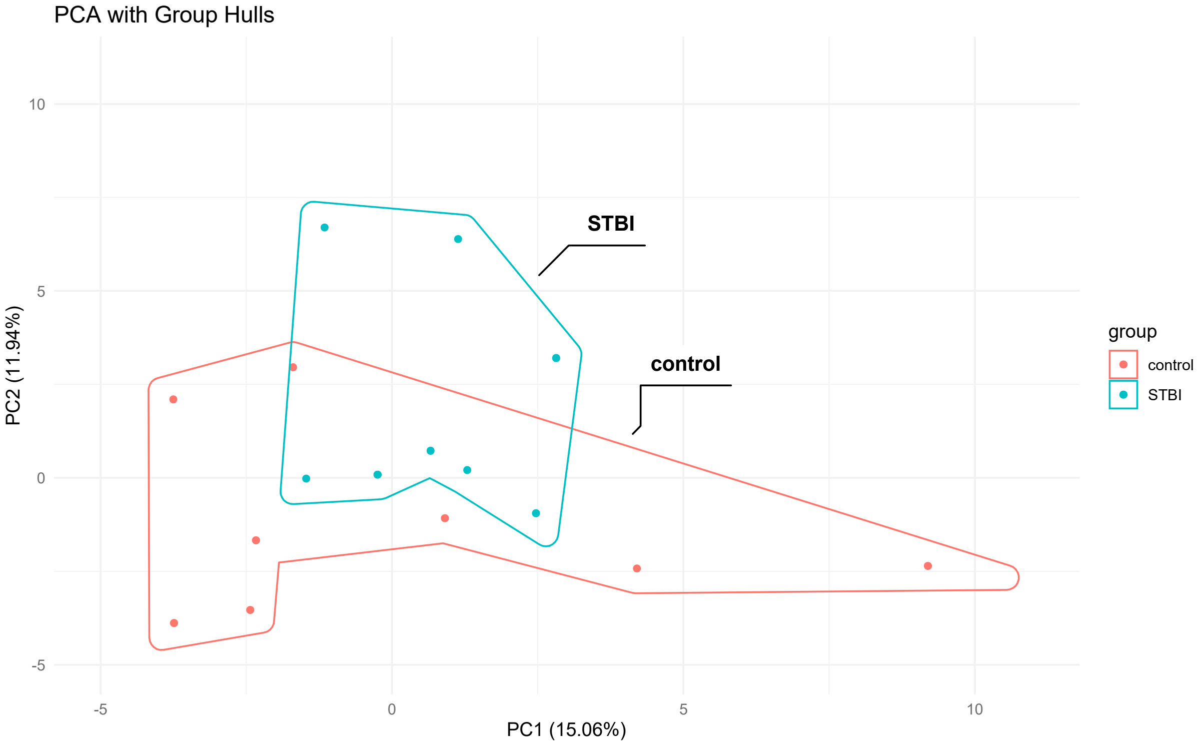

Figure S2: Principal Component Analysis (PCA) for the controls and TBI groups using Hulls plot.

Table S3. Statistics of sample gene expression

Sample	Exp gene	Min.	1st Qu.	Median	Mean	3rd Qu.	Max.	Sd.	Sum.
Control_1	995887	0	0.65	1.18	1.36	1.85	373.03	1.2	1356206
Control_2	1001940	0	0.7	1.21	1.38	1.85	305.94	1.16	1378003
Control_3	1011889	0	0.85	1.28	1.37	1.76	283.23	0.98	1383409
Control_4	447534	0	1.15	3.05	4.17	5.99	169.27	4.04	1868169
Control_5	580188	0	0.81	2.12	3.18	4.91	181.85	3.19	1843490
Control_6	1006817	0	0.73	1.21	1.35	1.8	240.03	1.02	1355063
Control_7	1009884	0	0.81	1.26	1.35	1.76	366.98	1.05	1358756
Control_8	709195	0	0.64	1.49	2.34	3.02	180.33	2.61	1661434
STBI_1	556549	0	0.52	2.22	3.39	5.26	177.24	3.67	1886389
STBI_2	860797	0	0.55	1.24	1.66	2.27	239.76	1.69	1424827
STBI_3	746105	0	0.58	1.39	2.06	2.68	228.53	2.33	1533325
STBI_4	609499	0	0.49	1.92	3	4.7	163.89	3.25	1828879
STBI_5	378701	0	0.8	4.04	5.39	7.88	155.01	5.82	2042238
STBI_6	436535	0	1.26	3.7	4.58	6.49	158.09	4.35	1999452
STBI_7	1005430	0	0.76	1.23	1.33	1.77	271.22	1.12	1342193
STBI_8	522885	0	0.82	2.52	3.6	5.41	167.54	3.65	1883612

Table S4. FPMK interval of gene expression

Sample	0-0.1 FI	0.1-0.3 FI	0.3-3.57 FI	3.57-15 FI	15-60 FI	>60 FI
Control_1	23883(2.40%)	71676(7.20%)	872069(87.57%)	28152(2.83%)	90(0.01%)	17(0.00%)
Control_2	18878(1.88%)	64143(6.40%)	892532(89.08%)	26268(2.62%)	101(0.01%)	18(0.00%)
Control_3	6886(0.68%)	32202(3.18%)	963900(95.26%)	8778(0.87%)	104(0.01%)	19(0.00%)
Control_4	21606(4.83%)	29068(6.50%)	196299(43.86%)	190662(42.60%)	9886(2.21%)	13(0.00%)
Control_5	25643(4.42%)	42256(7.28%)	317324(54.69%)	191433(32.99%)	3519(0.61%)	13(0.00%)
Control_6	15108(1.50%)	55040(5.47%)	919399(91.32%)	17176(1.71%)	76(0.01%)	18(0.00%)
Control_7	9082(0.90%)	38964(3.86%)	953613(94.43%)	8078(0.80%)	127(0.01%)	20(0.00%)
Control_8	34425(4.85%)	62184(8.77%)	471071(66.42%)	140309(19.78%)	1188(0.17%)	18(0.00%)
STBI_1	63914(11.48%)	47793(8.59%)	240974(43.30%)	196773(35.36%)	7083(1.27%)	12(0.00%)
STBI_2	48487(5.63%)	83417(9.69%)	642916(74.69%)	85753(9.96%)	206(0.02%)	18(0.00%)
STBI_3	41430(5.55%)	72025(9.65%)	513851(68.87%)	117862(15.80%)	921(0.12%)	16(0.00%)
STBI_4	69500(11.40%)	55844(9.16%)	288559(47.34%)	191752(31.46%)	3833(0.63%)	11(0.00%)
STBI_5	43216(11.41%)	28133(7.43%)	106733(28.18%)	176627(46.64%)	23956(6.33%)	36(0.01%)
STBI_6	33435(7.66%)	24420(5.59%)	155533(35.63%)	210044(48.12%)	13091(3.00%)	12(0.00%)
STBI_7	12031(1.20%)	47857(4.76%)	934424(92.94%)	10988(1.09%)	106(0.01%)	24(0.00%)
STBI_8	42624(8.15%)	35747(6.84%)	240044(45.91%)	197553(37.78%)	6906(1.32%)	11(0.00%)

Table S5. Gene Ontology Analysis for the differentially expressed genes.

GO Term	GO function	Genes	p-value
G-protein coupled receptor activity	molecular function	CXCR6;NPBWR2;NPY4R;OR10A6;OR14A2;OR6F1;OR1B1;OR1L1;OR2A4;OR4C13;OR4D1;OR4K13;OR51B2;OR52A5;OR5H2;SFRP2;TAAR6	0
olfactory receptor activity	molecular function	OR10A6;OR14A2;OR6F1;OR1B1;OR1L1;OR2A4;OR4C13;OR4D1;OR4K13;OR51B2;OR52A5;OR5H2	0
detection of chemical stimulus involved in sensory perception of smell	biological process	OR10A6;OR14A2;OR6F1;OR1B1;OR1L1;OR2A4;OR4C13;OR4D1;OR4K13;OR51B2;OR52A5;OR5H2	0
sensory perception of smell	biological process	OR10A6;OR14A2;OR6F1;OR1B1;OR1L1;OR2A4;OR4C13;OR4D1;OR4K13;OR51B2;OR52A5;OR5H2	0
G-protein coupled receptor signaling pathway	biological process	CXCR6;NPBWR2;NPY4R;OR10A6;OR14A2;OR6F1;OR1B1;OR1L1;OR2A4;OR4C13;OR4D1;OR4K13;OR51B2;OR52A5;OR5H2;SFRP2;TAAR6	0
detection of chemical stimulus involved in sensory perception	biological process	OR1B1;OR2A4;OR4C13;OR4D1;OR4K13	0
signal transducer activity	molecular function	CXCR6;NPBWR2;NPY4R;OR10A6;OR14A2;OR6F1;OR1B1;OR1L1;OR2A4;OR4C13;OR4D1;OR4K13;OR51B2;OR52A5;OR5H2;TAAR6	0
response to stimulus	biological process	OR10A6;OR14A2;OR6F1;OR1B1;OR1L1;OR2A4;OR4C13;OR4D1;OR4K13;OR51B2;OR52A5;OR5H2	0
elongin complex	cellular component	ELOA3D;ELOA3	0
transmembrane signaling receptor activity	molecular function	KLRF1;OR1B1;OR2A4;OR4C13;OR4D1;OR4K13	0

intermediate filament	cellular component	KRTAP10-12;KRTAP15-1;KRTAP19-5;KRTAP2-4;KRTAP7-1	0
negative regulation of intrinsic apoptotic signaling pathway in response to DNA damage	biological process	SFRP2;SNAI1	0
nuclear exosome (RNase complex	cellular component	EXOSC1	0
cytosolic large ribosomal subunit	cellular component	RPL10L;RPL23A;RPL23	0
translocation of peptides or proteins into host cell cytoplasm	biological process	TCP1	0
regulation of macrophage apoptotic process	biological process	TCP1	0
Bcl3-Bcl10 complex	cellular component	BCL3	0
ventral spinal cord interneuron fate determination	biological process	NKX2-2	0
aspartyl-tRNA aminoacylation	biological process	DARS	0
cellular response to growth hormone stimulus	biological process	STAR	0
negative regulation of cell differentiation involved in embryonic placenta development	biological process	SNAI1	0

negative regulation of dermatome development	biological process	SFRP2	0
type B pancreatic cell fate commitment	biological process	NKX2-2	0
thyroxine 5-deiodinase activity	molecular function	DIO3	0
positive regulation by host of symbiont catalytic activity	biological process	SUGT1	0
tRNA catabolic process	biological process	POP1	0
UDP-glucose 6-dehydrogenase activity	molecular function	UGDH	0
large ribosomal subunit rRNA binding	molecular function	RPL23	0
nuclear heterochromatin	cellular component	HIST1H1E;TCP1	0.01
ribosomal large subunit assembly	biological process	RPL10L;RPL23A	0.01
insecticide metabolic process	biological process	STAR	0.01
immunoglobulin production involved in immunoglobulin mediated immune response	biological process	GAPT	0.01

positive regulation of interleukin-10 biosynthetic process	biological process	BCL3	0.01
ribonuclease MRP activity	molecular function	POP1	0.01
regulation of midbrain dopaminergic neuron differentiation	NA	SFRP2	0.01
pancreatic PP cell fate commitment	biological process	NKX2-2	0.01
negative regulation of planar cell polarity pathway involved in axis elongation	biological process	SFRP2	0.01
Bcl3/NF-kappaB2 complex	cellular component	BCL3	0.01
aspartate-tRNA ligase activity	molecular function	DARS	0.01
pancreatic A cell fate commitment	biological process	NKX2-2	0.01
transcription factor activity, RNA polymerase III transcription factor binding	molecular function	BRF2	0.01
thyroid hormone catabolic process	biological process	DIO3	0.01
follicular dendritic cell differentiation	biological process	BCL3	0.01

positive regulation of Fc-gamma receptor signaling pathway involved in phagocytosis	NA	RAP1A	0.01
cysteine-type endopeptidase activity involved in apoptotic signaling pathway	molecular function	CASP10	0.01
3-oxoacid CoA-transferase activity	molecular function	OXCT2	0.01
CoA-transferase activity	molecular function	OXCT2	0.01
protein-DNA complex disassembly	biological process	RPL23	0.01
nucleosome	cellular component	HIST1H1E;HIST1H2AK;HIST1H3E	0.01
glycosaminoglycan metabolic process	biological process	B3GAT2;NDST1	0.01
signal transduction	biological process	CXCR6;GNRH2;NPBWR2;NPY4R;OR10A6;OR14A2;OR6F1;OR1B1;OR1L1;OR2A4;OR4C13;OR4D1;OR4K13;OR51B2;OR52A5;OR5H2;RAP1A;TAAR6	0.01
nuclear nucleosome	cellular component	HIST1H1E;HIST1H3E	0.01
keratinization	biological process	KRTAP10-12;KRTAP15-1;KRTAP19-5;KRTAP2-4	0.01
thyroxine 5'-deiodinase activity	molecular function	DIO3	0.01

positive regulation of bile acid biosynthetic process	biological process	STAR	0.01
UDP-glucose metabolic process	biological process	UGDH	0.01
meiotic DNA integrity checkpoint	biological process	HUS1B	0.01
cellular response to actinomycin D	biological process	RPL23	0.01
galactosylgalactosylxylosylprotein 3-beta-glucuronosyltransferase activity	molecular function	B3GAT2	0.01
phenol-containing compound metabolic process	biological process	STAR	0.01
development of primary male sexual characteristics	biological process	SFRP2	0.01
phthalate metabolic process	biological process	STAR	0.01
biphenyl metabolic process	biological process	STAR	0.01
XPC complex	cellular component	RAD23B	0.01
negative regulation of transport	biological process	DDI2;RSC1A1	0.01

N-acetylglucosamine deacetylase activity	molecular function	NDST1	0.01
cellular response to luteinizing hormone stimulus	biological process	STAR	0.01
positive regulation of bone development	biological process	TMEM119	0.01
DNA-templated transcriptional preinitiation complex assembly	biological process	BRF2	0.01
regulation of establishment of planar polarity	biological process	SFRP2	0.01
C-X-C chemokine binding	molecular function	CXCR6	0.01
spinal cord oligodendrocyte cell fate specification	biological process	NKX2-2	0.01
UDP-glucuronate biosynthetic process	biological process	UGDH	0.01
MHC class I receptor activity	molecular function	KLRF1	0.01
circadian sleep/wake cycle, REM sleep	biological process	STAR	0.01
spinal cord oligodendrocyte cell differentiation	biological process	NKX2-2	0.01

glycosaminoglycan biosynthetic process	biological process	NDST1;UGDH	0.02
rRNA binding	molecular function	RPL23A;SBDS	0.02
nucleotide-excision repair	biological process	HUS1B;RAD23B	0.02
nuclear-transcribed mRNA catabolic process, nonsense-mediated decay	biological process	PNRC1;RPL23A;RPL23	0.02
scaRNA localization to Cajal body	NA	TCP1	0.02
dibenzo-p-dioxin metabolic process	biological process	STAR	0.02
T-helper 2 cell differentiation	biological process	BCL3	0.02
ketone body catabolic process	biological process	OXCT2	0.02
negative regulation of vitamin D biosynthetic process	biological process	SNAI1	0.02
extracellular matrix protein binding	molecular function	CLEC14A	0.02
polysaccharide biosynthetic process	biological process	NDST1	0.02

sclerotome development	biological process	SFRP2	0.02
Wnt signaling pathway involved in somitogenesis	biological process	SFRP2	0.02
response to stilbenoid	biological process	LY6D	0.02
dATP binding	molecular function	HIST1H1E	0.02
negative regulation of synaptic vesicle exocytosis	biological process	RAP1A	0.02
regulation of NF-kappaB import into nucleus	biological process	BCL3	0.02
negative regulation of interleukin-8 biosynthetic process	biological process	BCL3	0.02
RNA polymerase III type 3 promoter DNA binding	molecular function	BRF2	0.02
regulation of cell junction assembly	biological process	RAP1A	0.02
death effector domain binding	molecular function	CASP10	0.02
negative regulation of mesodermal cell fate specification	biological process	SFRP2	0.02

humoral immune response mediated by circulating immunoglobulin	biological process	BCL3	0.02

Table S6. KEGG pathway analysis for the differentially expressed genes.

KEGG Pathway ID	Pathway	Genes	p-value
ko04740	Olfactory transduction	OR10A6;OR14A2;OR6F1;OR1B1;OR1L1;OR2A4;OR4C13;OR4D1;OR4K13;OR51B2;OR52A5;OR5H2	0
ko04972	Pancreatic secretion	AC040162.1;CTRL;PSMB10;CPA1;RAP1A	0.02
ko03010	Ribosome	RPL10L;RPL23A;RPL23	0.05
ko00072	Synthesis and degradation of ketone bodies	OXCT2	0.06
ko04622	RIG-I-like receptor signaling pathway	CASP10;IFNA8	0.07
ko03008	Ribosome biogenesis in eukaryotes	POP1;SBDS	0.09
ko04974	Protein digestion and absorption	AC040162.1;CTRL;PSMB10;CPA1	0.11
ko00770	Pantothenate and CoA biosynthesis	PANK3;SLC2A3P1	0.11
ko00053	Ascorbate and aldarate metabolism	UGDH	0.11
ko00515	Mannose type O-glycan biosyntheis	B3GAT2	0.14
ko00534	Glycosaminoglycan biosynthesis - heparan sulfate / heparin	NDST1	0.15
ko04950	Maturity onset diabetes of the young	NKX2-2	0.15
ko00040	Pentose and glucuronate interconversions	UGDH	0.15
ko04668	TNF signaling pathway	BCL3;CASP10	0.15
ko00650	Butanoate metabolism	OXCT2	0.16
ko05322	Systemic lupus erythematosus	HIST1H2AK;HIST1H3E	0.18
ko05161	Hepatitis B	CASP10;IFNA8	0.23
ko00970	Aminoacyl-tRNA biosynthesis	DARS	0.25
ko03420	Nucleotide excision repair	RAD23B	0.25

ko03050	Proteasome	AC040162.1;CTRL;PSMB10	0.25
ko00520	Amino sugar and nucleotide sugar metabolism	UGDH	0.26
ko00280	Valine, leucine and isoleucine degradation	OXCT2	0.26
ko05320	Autoimmune thyroid disease	IFNA8	0.26
ko04217	Necroptosis	HIST1H2AK;IFNA8	0.27
ko04979	Cholesterol metabolism	STAR	0.27
ko04913	Ovarian Steroidogenesis	STAR	0.27
ko04621	NOD-like receptor signaling pathway	IFNA8;SUGT1	0.28
ko05034	Alcoholism	HIST1H2AK;HIST1H3E	0.3
ko04080	Neuroactive ligand-receptor interaction	NPBWR2;NPY4R;TAAR6	0.3
ko05152	Tuberculosis	CASP10;IFNA8	0.3
ko05202	Transcriptional misregulation in cancers	HIST1H3E;REL	0.32
ko04062	Chemokine signaling pathway	CXCR6;RAP1A	0.32
ko04623	Cytosolic DNA-sensing pathway	IFNA8	0.33
ko04927	Cortisol synthesis and secretion	STAR	0.33
ko04720	Long-term potentiation	RAP1A	0.35
ko05211	Renal cell carcinoma	RAP1A	0.35
ko04520	Adherens junction	SNAI1	0.37
ko03018	RNA degradation	EXOSC1	0.4
ko04014	Ras signaling pathway	RAP1A;REL	0.43
ko04912	GnRH signaling pathway	GNRH2	0.44
ko04925	Aldosterone synthesis and secretion	STAR	0.46
ko04620	Toll-like receptor signaling pathway	IFNA8	0.48
ko04060	Cytokine-cytokine receptor interaction	CXCR6;IFNA8	0.51

ko04670	Leukocyte transendothelial migration	RAP1A	0.51
ko04919	Thyroid hormone signaling pathway	DIO3	0.53
ko04722	Neurotrophin signaling pathway	RAP1A	0.53
ko04611	Platelet activation	RAP1A	0.54
ko04650	Natural killer cell mediated cytotoxicity	IFNA8	0.55
ko05160	Hepatitis C	IFNA8	0.57
ko05162	Measles	IFNA8	0.58
ko04210	Apoptosis	CASP10	0.59
ko04310	Wnt signaling pathway	SFRP2	0.6
ko04630	Jak-STAT signaling pathway	IFNA8	0.65
ko04141	Protein processing in endoplasmic reticulum	RAD23B	0.66
ko05164	Influenza A	IFNA8	0.66
ko04530	Tight junction	RAP1A	0.66
ko03013	RNA transport	POP1	0.67
ko05168	Herpes simplex infection	IFNA8	0.69
ko05167	Kaposi's sarcoma-associated herpesvirus infection	IFNA8	0.7
ko04024	cAMP signaling pathway	RAP1A	0.72
ko05203	Viral carcinogenesis	REL	0.72
ko04510	Focal adhesion	RAP1A	0.72
ko04015	Rap1 signaling pathway	RAP1A	0.74
ko04010	MAPK signaling pathway	RAP1A	0.85
ko05165	Human papillomavirus infection	IFNA8	0.87
ko04151	PI3K-Akt signaling pathway	IFNA8	0.89
ko05200	Pathways in cancer	IFNA8	0.97
